# Self-Paced Reaching after Stroke: A Quantitative Assessment of Longitudinal and Directional Sensitivity Using the H-Man Planar Robot for Upper Limb Neurorehabilitation

**DOI:** 10.3389/fnins.2016.00477

**Published:** 2016-10-25

**Authors:** Asif Hussain, Aamani Budhota, Charmayne Mary Lee Hughes, Wayne D. Dailey, Deshmukh A. Vishwanath, Christopher W. K. Kuah, Lester H. L. Yam, Yong J. Loh, Liming Xiang, Karen S. G. Chua, Etienne Burdet, Domenico Campolo

**Affiliations:** ^1^Robotics Research Centre, School of Mechanical and Aerospace Engineering, Nanyang Technological UniversitySingapore, Singapore; ^2^Interdisciplinary Graduate School, Nanyang Technological UniversitySingapore, Singapore; ^3^Department of Kinesiology, San Francisco State UniversitySan Francisco, CA, USA; ^4^Health Equity Institute, San Francisco State UniversitySan Francisco, CA, USA; ^5^Department of Bioengineering, Imperial College of Science, Technology and MedicineLondon, UK; ^6^Centre for Advanced Rehabilitation Therapeutics, Tan Tock Seng Hospital Rehabilitation CentreSingapore, Singapore; ^7^School of Physical and Mathematical Sciences, Nanyang Technological UniversitySingapore, Singapore

**Keywords:** stroke, neurorehabilitation, robotic assessment, stroke rehabilitation, sensorimotor assessment

## Abstract

Technology aided measures offer a sensitive, accurate and time-efficient approach for the assessment of sensorimotor function after neurological insult compared to standard clinical assessments. This study investigated the sensitivity of robotic measures to capture differences in planar reaching movements as a function of neurological status (stroke, healthy), direction (front, ipsilateral, contralateral), movement segment (outbound, inbound), and time (baseline, post-training, 2-week follow-up) using a planar, two-degrees of freedom, robotic-manipulator (H-Man). Twelve chronic stroke (age: 55 ± 10.0 years, 5 female, 7 male, time since stroke: 11.2 ± 6.0 months) and nine aged-matched healthy participants (age: 53 ± 4.3 years, 5 female, 4 male) participated in this study. Both healthy and stroke participants performed planar reaching movements in contralateral, ipsilateral and front directions with the H-Man, and the robotic measures, spectral arc length (SAL), normalized time to peak velocities (*T*_*peakN*_), and root-mean square error (RMSE) were evaluated. Healthy participants went through a one-off session of assessment to investigate the baseline. Stroke participants completed a 2-week intensive robotic training plus standard arm therapy (8 × 90 min sessions). Motor function for stroke participants was evaluated prior to training (baseline, week-0), immediately following training (post-training, week-2), and 2-weeks after training (follow-up, week-4) using robotic assessment and the clinical measures Fugl-Meyer Assessment (FMA), Activity-Research-Arm Test (ARAT), and grip-strength. Robotic assessments were able to capture differences due to neurological status, movement direction, and movement segment. Movements performed by stroke participants were less-smooth, featured longer *T*_*peakN*_, and larger RMSE values, compared to healthy controls. Significant movement direction differences were observed, with improved reaching performance for the front, compared to ipsilateral and contralateral movement directions. There were group differences depending on movement segment. Outbound reaching movements were smoother and featured longer *T*_*peakN*_ values than inbound movements for control participants, whereas SAL, *T*_*peakN*_, and RMSE values were similar regardless of movement segment for stroke patients. Significant change in performance was observed between initial and post-assessments using H-Man in stroke participants, compared to conventional scales which showed no significant difference. Results of the study indicate the potential of H-Man as a sensitive tool for tracking changes in performance compared to ordinal scales (i.e., FM, ARAT).

## Introduction

The rehabilitation of neurological disorders such as stroke and cerebral palsy is a labor -intensive process that requires daily one-on-one interactions with therapists. The significant burden placed on the health care providers and the overall health care system have stimulated particular interest in technology assisted systems for neurorehabilitation (Maciejasz et al., 2014), with the underlying objective of decreasing the workload of the therapist and to facilitate training with minimal supervision at an affordable cost. A significant amount of this work has focused on the development of robotic devices to train upper extremity (UE) task-related movements (Riener et al., 2005; Prange et al., 2006; Brewer et al., 2007; Balasubramanian et al., 2010). The advantages of robot-assisted therapy include the ability to actively assist or resist human motions, to acquire accurate measurements of the dynamic and kinematic performance of participants during training using integrated sensors, and to administer repetitive task-specific training with limited supervision from a therapist. To date, clinical studies have shown that robot-assisted therapy of the UE is at least as effective as conventional rehabilitation therapy in terms of reducing motor impairments over a short-term period (Prange et al., 2006; Kwakkel et al., 2008; Lo et al., 2010; Norouzi-Gheidari et al., 2012) and thus can effectively complement conventional therapy. Although conventional therapy, itself is not very productive/ efficient, Duncan et al. reported that only 33–70% of the stroke patients recover useful arm ability, and initial paresis severity remains the best predictor of arm function recovery over 6 months (Duncan et al., 1992; Huang and Krakauer, 2009). It is possible that the limited recovery success for UE dysfunction after stroke is hampered by the limited amount of training offered to the affected population. As such, increasing the frequency and intensity of training could significantly improve performance (Harvey, 2009). However, an arguably equal, if not more important factor contributing to this limited improvement can be attributed to the partial understanding and incomplete assessment of the disability itself, which in technology intervention systems has been explored less thoroughly. Clear knowledge of the level of sensorimotor deficits is required for devising a comprehensive and efficient training regime (Balasubramanian et al., 2012a).

Conventionally, assessment of motor functions is carried out by therapists by means of ordinal clinical scales to examine specific aspects of a subject's motor behavior and devise an appropriate treatment strategy accordingly (Fugl-Meyer et al., 1974; Lyle, 1981; Gladstone et al., 2002). For example, the Action Research Arm Test (ARAT) scores performance on various tasks using a 4-point scale, where 0 indicates no movement and 3 indicates the task is completed with normal performance (Lyle, 1981). Although the ARAT and other post-stroke motor assessments are widely accepted and have high test-retest and interrater reliability, their reliance on ordinal scoring renders them insensitive to subtle differences in deficit and changes over the rehabilitation lifespan. Furthermore, the additional time required to perform manual assessment discourages their regular use in clinical practice to track and understand motor recovery in the affected population.

It is apparent that stroke rehabilitation would benefit if clinicians had a complete understanding of the specific sensorimotor deficits exhibited by the patient (Balasubramanian et al., 2012a). Robotic technology has the potential to augment the assessment process by using integrated sensors to record continuous, high-resolution data. These sensory measurements are collected during normal use of the system and do not require additional time for a discrete assessment protocol. These systems are (semi-) autonomous, potentially more objective than functional assessments, and less prone to human error/subjectivity (Bosecker et al., 2010; Lambercy et al., 2010). However, this form of assessment has yet to be fully established and validated when compared to the gold standards, and is expensive due to the high cost of (most) robotic systems for use in standard clinical practice.

At Nanyang Technological University (NTU) we have designed a novel low-cost, planar, table-top robot for decentralized neurorehabilitation (hereafter called H-Man) (Campolo et al., 2014; Hussain et al., 2015a). It can benefit participants with limited access to a therapist for rehabilitation, and with properly validated assessment protocols, can provide continuous updates about motor progress to the patient, their caregivers, and the therapy team. In this study, we evaluated the ability of the H-Man to detect differences in planar self-paced reaching as a function of neurological status (stroke, age-matched healthy control), direction (front, ipsilateral, contralateral), and movement segment (outbound, inbound). In addition, we investigated the longitudinal sensitivity of these performance metrics to capture motor performance changes in stroke patients, and examined the relationship between robotic measures and conventional scales. Multiple studies have previously addressed variations in performance metrics on workspace. However, due to variations in protocols/task definitions (for example point to point vs. path reaching, free reaching vs. supported movements) and varying outcomes, the reliability, and validity of reaching movements as measures of upper limb motor functionality is still limited (Levin, 1996; Archambault et al., 1999; Kamper et al., 2002; Sukal et al., 2007). Moreover, most of these studies focus on developing relations to clinical scales and/or inter-relationships between performance metrics. In this paper, we focus on a more fundamental question: the distribution/variation of performance outcomes within a control group and across stroke participants for different directions, and for different segments of movements [outbound movements (i.e., away from the body) and inbound movement (i.e., toward the body)]. Multiple papers briefly address this question but not as a major focus of study for-example, Kamper et al. and Levin presented studies on free reaching in 3D and planar supported reaching tasks, respectively (Levin, 1996; Kamper et al., 2002), which showed modest variations across directions but pre-dominantly focused on results (of all directions) to establish relationships with performance matrices and or clinical scales. Here, we report variations for all directions and performance matrices, for both control and stroke participants, along with comparisons between inbound and outbound movement segments. These results help build a clearer understanding of the characteristics of reaching movements and how they differ across stroke and healthy participants. Further, we also show the sensitivity of selected performance measures compared to clinical scales by analysing longitudinal changes in metrics by assessing performance over a 2-week period, which adds weight to the potential of the H-Man as an effective assessment tool.

## Methods

Prior to subject recruitment, ethical approvals were obtained from the Domain Specific Institutional Review Board (IRB) of the National Healthcare Group (NHG), Singapore. All subjects gave written informed consent prior to screening procedures and recruitment (clinical-trial ID: NCT02188628—clinicaltrials.gov). The study was conducted in accordance with the declaration of Helsinki.

### Study design: prospective open label clinical feasibility trial

#### Participants

Study inclusion criteria were first ever clinical stroke (ischaemic or hemorrhagic) confirmed by brain imaging, post-stroke duration of 3–24 months, with shoulder abduction and elbow flexion greater or equal to 3/5 on the Medical Research Council scale for muscle strength, and a Fugl-Meyer Upper Extremity Motor Assessment (FMA) score of 20–50 or pre-dominant motor ataxia or incoordination (FMA > 50). Participants were excluded if they had any non-stroke related arm impairment, moderate arm spasticity as indicated by the Modified Ashworth Scale (>2), moderate shoulder pain (VAS > 5/10), visual impairment (hemianopia), visual spatial neglect, and/or cognitive impairments [Mini Mental State Exam (MMSE) < 26/28].

Nine neurologically healthy individuals served as aged matched control participants (mean age: 53 ± 4.3, 4 male, 5 female).

#### Apparatus and assessment procedure

**Figure 1:**
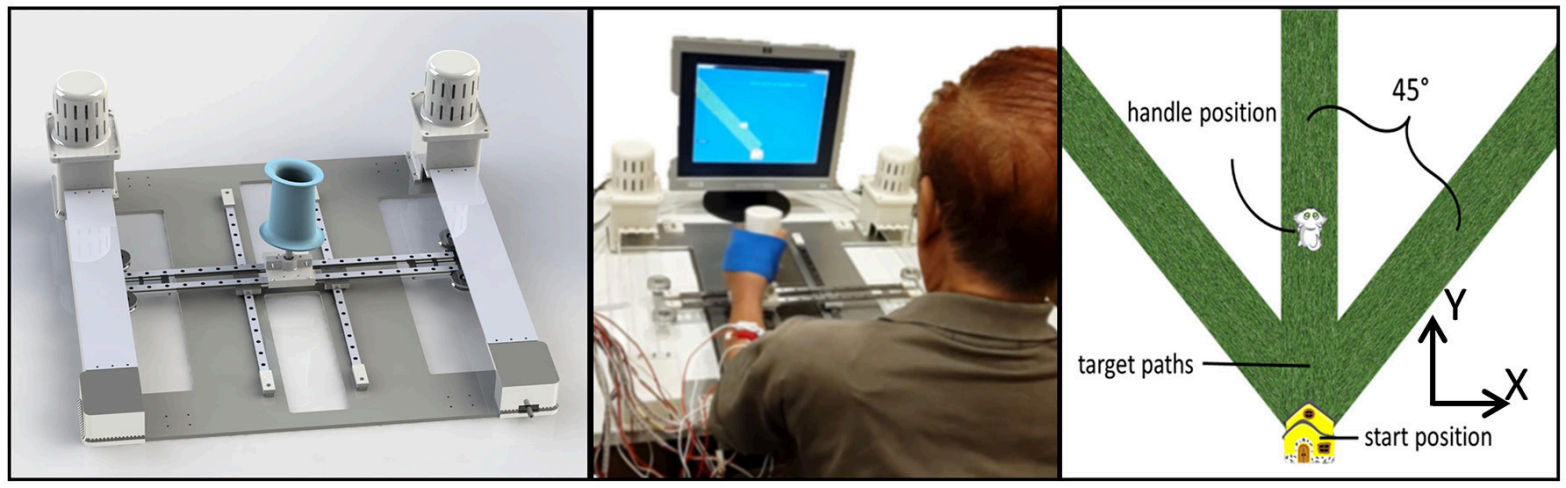
**(Left) Cad model of H-Man, a compact robot designed for the rehabilitation/training of the upper-limb. (Middle)** A Stroke Participant using H-Man in Hospital. **(Right)** Representation of visual stimuli used for the assessment using H-Man.

The experimental apparatus used for the study is shown in Figure 1. H-Man is a compact robot designed for the rehabilitation/training of planar arm movements (Campolo et al., 2014). It has an H-shaped cable-driven differential mechanism. This mechanism is advantageous because of its homogeneous workspace, lightweight profile, and intrinsic safety of use (Campolo et al., 2014). H-Man, can provide forces of up to 30 N at the end-effector (handle) in any specified direction in a planar workspace to assist or resist the motion of the user, and can be easily built using off the shelf components. For further information, the reader is referred to Campolo et al. (2014) for a detailed description and the characteristic parameters of H-Man, along with references to works in which H-Man has been studied with control and stroke participants (Campolo et al., 2014; Hussain et al., 2015a,b).

H-Man was placed in front of the subject on a fixed table, behind which a 43 cm flat screen monitor (Sync Master 943T, Samsung) displayed a virtual representation of the workspace/task and provided visual feedback throughout the experiment. The visual stimuli consisted of a start position (virtually represented as a house), the required movement path (virtually represented as a grass path), the cursor controlled by the H-Man handle (virtually represented as a cat), and the task instructions.

Participants were seated in a height-adjustable chair in front of the table, so that the center of the sternum was aligned with the handle of the H-Man robot, and the elbow bent at 90°. At the start of each trial a movement path (i.e., grass path) was visually displayed on the computer monitor in the contralateral, ipsilateral and sagittal plane direction (angles of ±45° and 0° from the vertical axis) and participants used the robot handle to move the virtual object (i.e., the cat) as far as possible along the movement path. Instructions emphasized that after reaching the maximum distance they should hold that position for 3 s, after which the participants were allowed to bring the virtual object back to the start position (i.e., the house) while remaining on the movement path. No physical trunk restraint was used during the experiment in order to assess the natural performance of the participant. However, participants were instructed to limit their trunk movements while performing the task.

Stroke participants performed 36 trials in total (12 in each direction in randomized order) and control participants completed 15 trials per direction (randomized order) for a total of 45 trials for each subject. All robotic assessment sessions were supervised by an occupational therapist and an engineer. Duration of H-man session was 1 h followed by 30 min of conventional occupational therapy directed toward neuro-facilitation, active range of motion exercises, self-care activities of daily living and home exercise programme. The intensity of training was 4 times per week for consecutive 2 weeks (total of 8 sessions). The conventional therapy included passive mobilization and active-assisted approaches based on Neurodevelopmental Technique to enhance normal movement patterns, repetitive task specific training for functional reach training (Howle, 2002; Langhorne et al., 2011) and use of upper limb inclined board and motorized arm bike.

Patient outcome assessments were performed at baseline (week 0), post-training (week 2), and follow-up (2 weeks post-training) by independent senior occupational therapist. Primary outcome measures included the FMA, ARAT and grip strength assessed via DynEx dynamometer (Fugl-Meyer et al., 1974; Hsieh et al., 1998). Adverse events such as increased pain (visual analog scale 0–100), increased arm spasticity (Modified Ashworth Scale (0–4), and dropout rate were also measured.

### Data analysis

The end-effector position data obtained from encoders located on H-Man and the derived velocity (from the position) was filtered using a low pass filter (Butterworth: 6th order, cut-off Fc: 20 Hz, sampling rate Fs: 1000 Hz). For each trial, the time series was divided into an outward and inward movement segment by analysing the filtered distance and velocity profile along with the state of the task defined by the game (visual display).

The outward movement segment was defined as movement from the instant the distance crosses the base positon (2 cm) and ends at 95% of the maximum distance reached. It was followed by the rest phase where the distance covered is above 95%. The inward movement is the return phase of the movement, starting from instant when the distance to the base station is below 95% of the maximum distance, to the instant the subject reaches the base station. The segmented movement is further refined (to remove any abnormalities) by analysing the velocity profile. For both outbound and inbound movements, the onset time (*T*_*onset*_) is the time instant when the tangential velocity exceeds the 5% of the maximum velocity (*V*_*peak*_) and offset time (*T*_*offset*_) is the time velocity drops below the threshold of 5% of the maximum velocity (*V*_*peak*_).

The segmented kinematic information from all directions was used in offline processing to calculate multiple task performance indices adopted from literature (Balasubramanian et al., 2012a). Out of these, three dominant measures corresponding to smoothness, temporal (task) efficiency, and task error were selected, namely spectral-arc length, time to peak Velocity (distance normalized) and deviation fit root mean square error. The criteria for measure selection emphasized the significance of these measures in the literature and observation of results from the clinical trials as representative of a trend, keeping in consideration the sensitivity (significant differences between groups/sessions-ability to detect change) of each measure. For-example, with respect to smoothness measure spectral arc length (SAL), was selected due to its strong correlation with Log-jerk and number of peak velocity (in our task) and it has been demonstrated as a more reliable measure for measuring smoothness (Balasubramanian et al., 2012b). Each measure is briefly discussed below:

#### Spectral arc length (SAL)

The smoothness of each reaching motion was assessed using the SAL metric developed in Balasubramanian et al. (2012b). SAL is a dimensionless measure of the length of the frequency spectrum curve of a speed profile over the bandwidth appropriate for the action. It is defined as follows:

$$\begin{array}{l} \eta_{sal}=-\int_{0}^{\omega_{c}}\sqrt{{\left(\frac{1}{\omega_{c}}\right)}^{2}+{\left(\frac{d\hat{V}\left(\omega \right)}{d\omega }\right)}^{2}}d\omega \end{array}$$

where [0, ω_*c*_] is the frequency band of interest (typically up to 20 Hz for normal human movement) and the Fourier magnitude spectrum of the velocity signal.. is given by:

$$\begin{array}{l} \hat{V}\left(\omega \right)=\frac{V\left(\omega \right)}{V\left(0\right)} \end{array}$$

#### Normalized time to peak velocity

Time taken to reach peak velocity (*T*_*peak*_) is given by the difference between the time at which *V*_*peak*_ is reached and the time at which velocity first exceeds 5% of peak velocity (*T*_*onset*_).

$$\begin{array}{l} T_{peak}=T\;\left(V_{peak}\right)-T_{onset}\\ Normalized\;Time\;to\;peak\;Velocity\;\left(T_{peakN}\right)=\frac{T_{peak}}{D_{max}} \end{array}$$

Where *D*_*max*_ is the total distance covered during the trial.

#### Root mean square error (RMSE)

RMSE assesses the deviation of the observed path from the straight line fitted between the starting point and final position of the handle using linear regression, and is given by:

$$\begin{array}{l} RMSE=\sqrt{\frac{\sum_{i=1}^{n}{\left(\hat{y_{i}}-y_{i}\right)}^{2}}{n}} \end{array}$$

where *y* and $\hat{y}$ denote the observed and the predicted data points(obtained from linear regression), respectively.

### Statistical analysis

The specific aims of the study were to examine differences in planar reaching as a function of (1) neurological status (stroke patient, age-matched controls), (2) direction [contralateral, ipsilateral, and sagittal plane (front)], and movement segment (inbound or outbound), and (3) time (pre-training, post-training, and 2-week follow-up). Given that some participants performed the task with the left hand (dominant hand for control and or affected hand for stroke participants), while others performed the task with the right hand, the independent variable *movement direction* was coded with respect to an egocentric frame of reference. As such, when movements were performed with the left hand, ipsilateral refers to the −45°movement direction, contralateral refers to the 45° movement direction, and front refers to the 0° movement direction. When movements were performed with the right hand, ipsilateral, front and contralateral correspond to the +45°, 0°, and −45° movement directions, respectively.

**Figure 2:**
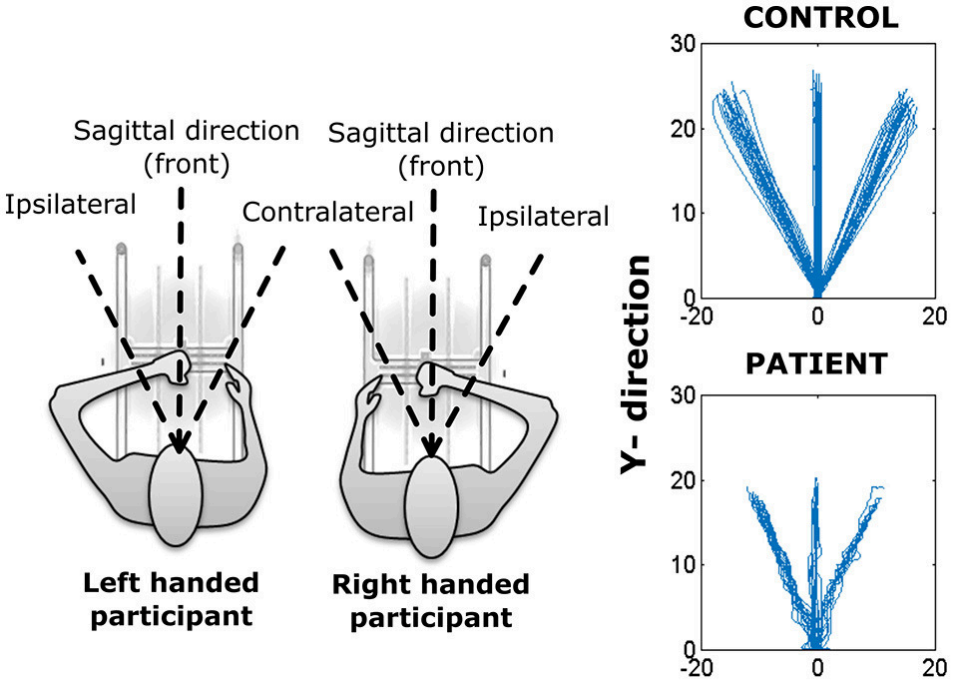
**(Left) Representation of H-Man use with left and right hand. (Right)** Reaching trajectories of a healthy (control) participant and a stroke participant.

For the remainder of this paper, we will use the term movement segment/phase to correspond to outbound and inbound movements of a trial while movement direction will refer to one of the three directions (contralateral, ipsilateral, and front) selected for that segment (inbound/outbound) of movement (see Figure 2).

Based on this convention and aforementioned aims we highlighted following questions and analyzed them individually for each specific measure using non-parametric tests (discussed below), since normality condition was not satisfied in multiple cases (Kolmogorov-Smirnov (K-S) test).

#### Intra-group: directional differences between control and stroke participants

Kruskal-Wallis (KW) analysis, a rank-based non-parametric test was used on the data of healthy (control) participants to investigate the difference between the three different directions. The analysis was carried out for both inbound and outbound segments of movements separately. Significant main effects were compared using Tukey's honest significant difference test (HSD) *post-hoc* analysis. Further, the Mann–Whitney *U*-test (also referred to as Wilcoxon rank-sum test-RS test) was employed to compare inbound vs. outbound movement segments (separately for each movement direction).

#### Inter-group: directional differences between control and stroke participants

Rank Sum-test (RS-test) was used to compare differences between control and stroke participants for each movement direction, i.e., front direction of control participants was compared with front direction of stroke participants. The analysis was carried out for both inwards and outwards movement segments

#### Over-time: differences over sessions in stroke patients

KW test was used to assess the performance of stroke participants across the three assessment time points (baseline, post-training, follow-up), separately for each movement segment. Significant main effects were compared using Tukey's HSD *post-hoc* analysis.

## Results

Demographic and clinical data are provided in Table 1. Twelve stroke patients (mean age: 55, *SD*: 10.0, 7 males, 5 females, mean time since stroke: 11.2 months, *SD*: 6.0) participated in the study between January and July 2015. Seven patients had an intracerebral hemorrhage (mean age: 49.3, *SD*: 4.3, mean time since stroke: 9.6 months, *SD*: 2.6), and five patients had an infarct (ischaemic stroke, mean age: 58.0, *SD*: 11.3, mean time since stroke: 12.3 months, *SD*: 7.0). Seven patients had hemiplegia of the right arm and five patients had hemiplegia of the left arm.

**Table 1:** **Baseline demographic and clinical characteristics of 12 stroke participants (Stroke type: M, Male; F, Female; Stroke type: ICH, intracerebral hemorrhage; IS, ischaemic stroke; Affected arm: R, Right; L, Left)**.

**Subject**	**Age (years)**	**Gender**	**Duration post-stroke (months)**	**Stroke type**	**Affected arm**	**FMA**	**ARAT**	**Localization of stroke**
1	54	M	22	IS	R	55^*^	30	Lacunar stroke
2	57	M	6	IS	R	28	6	Lacunar stroke
3	75	M	4	IS	L	48	49	Post-circulation
4	51	F	7	ICH	R	29	8	Basal ganglia/thalamus
5	66	M	6	IS	R	64^*^	56	Lacunar stroke
6	57	F	7	IS	R	46	20	Post-circulation
7	52	M	20	ICH	R	30	19	Basal ganglia/thalamus
8	52	F	5	ICH	L	43	16	Basal ganglia/thalamus
9	38	F	16	IS	R	29	7	Total anterior circulation stroke
10	45	M	13	ICH	L	45	25	Basal ganglia/thalamus
11	56	F	11	ICH	R	43	26	Basal ganglia/thalamus
12	67	M	19	IS	L	20	3	Partial anterior circulation stroke

* Indicates pre-dominant motor ataxia.

Analysis of clinical outcomes revealed no noticeable change in total FMA (0.58 ± 2.82), *p* > 0.48), ARAT (2.25 ± 3.8, *p* > 0.066) scores after 2 weeks of robotic training (post-training). Furthermore, there was also no change in arm function at 2-week follow-up assessment when compared to the baseline assessment (FMA: −0.99±2.92; ARAT: 2.67 ± 4.37, both *p*'s > 0.05) (Table 2).

**Table 2:** **Summary of changes in clinical outcomes**.

**Outcomes**	**Mean (*SD*)**	***P*-value**
ARAT (week 0–2)	2.25 (3.8)	0.066
ARAT (week 0–4)	2.67 (4.37)	0.058
Grip strength (KgF) (week 0–2)	0.99 (2.61)	0.217
Grip strength (KgF) (week 0–4)	0.98 (1.98)	0.12
FMA total (week 0–2)	0.58 (2.82)	0.487
FMA total (week 0–4)	−0.33 (2.92)	0.698

Post-training grip strength values (week 2) were similar to baseline (mean difference = 0.99 ± 2.61, *p* > 0.2) and there was no noticeable difference at 2-week follow-up (mean difference = 0.98 ± 1.98, *p* > 0.1). This limited improvement was expected due to relatively short duration (2 weeks) of training for chronic stroke participants.

No increases in pain, spasticity or other adverse events were reported by participants. However, 9 out of 12 subjects reported subjective gains in arm use and a positive training experience with the H-man robot.

### Differences between movement directions and segments within groups: healthy participants

#### Spectral arc length (SAL)

Overall, the outbound segment of movements (−2.2730±0.23) were significantly smoother when compared to corresponding inbound segments (−2.42±0.37) of movements, independent of movement direction (all *p*'s < 0.004, RS-test) (Figure 2).

There was a significant movement direction difference in SAL values for outbound movements (contralateral = −2.27±0.29, ipsilateral = −2.35±0.21, front = −2.21±0.19), *p* < 0.001. *Post-hoc* Tukey's HSD test indicated that SAL values were smaller for outbound movements in the front direction compared to the contralateral movement direction but was not significantly different from ipsilateral movement direction. Similarly, there was a significant movement direction difference in SAL values for inbound movements (contralateral = −2.43±0.41, ipsilateral = −2.48±0.37, front = −2.35±0.34), *p* < 0.02. *Post-hoc* Tukey's HSD test indicated that the frontal direction resulted in smoother inbound movements overall.

#### Normalized time to peak velocity

Time to peak velocity value were significantly shorter for inbound movement (3.87 ± 2.93) compared to outbound (5.25 ± 3.31), regardless of the movement direction (i.e., ipsilateral, contralateral, or front direction) (all *p*'s < 0.001).

Similar to SAL, there was a significant main effect of movement direction for outbound movements, *p* < 0.001 (**Figure 4**). *Post-hoc* Tukey's HSD test revealed shorter time to peak velocity values for outbound movements to the front (contralateral = 6.12 ± 4.9, ipsilateral = −5.11±2.86, front = −4.53±2.15) compared to the contralateral movement direction. In contrast, for inbound segment of movements, no significant effect of direction was observed (contralateral = 3.98 ± 3.52, ipsilateral = 3.70 ± 3.02, front = 3.93 ± 2.27, (*p* > 0.169).

#### Root mean square error

There was a significant main effect of movement segment for the front (*p* < 0.02) and ipsilateral (*p* < 0.001) direction, but not the contralateral direction (*p* > 0.1). RMSE values were larger for the front direction, for both the outbound (front = 1.56 ± 0.80, contralateral = 0.23 ± 0.15, ipsilateral = 0.21 ± 0.09) and inbound movement segments (front = 1.29 ± 0.72, contralateral = 0.38 ± 0.20, ipsilateral = 0.31 ± 0.12), as verified by Tukey's HSD test (both *p*'s < 0.001).

### Differences between movement directions and segments within groups: stroke patients

#### Spectral arc length

Within stroke participants, SAL values were similar regardless of movement segment (*p* > 0.1). However, there was a significant difference between movement directions within each movement segment (all *p*'s < 0.01). For outbound movements *post-hoc* Tukey's HSD test revealed there were significant differences in SAL between front and ipsilateral movement directions (front = −3.72±1.83, ipsilateral = −4.52± 2.62), *p* < 0.001. For inbound movements, *post-hoc* analysis indicated significant differences between front and contralateral movement directions (front = −3.64±1.71, contralateral = −4.48±2.66), *p* < 0.001.

#### Normalized time to peak velocity

Time to peak velocity was similar for outbound and inbound movement segments (16.06 ± 21.98; 14.25 ± 24.88, respectively), all *p*'s > 0.1.

**Figure 3:**
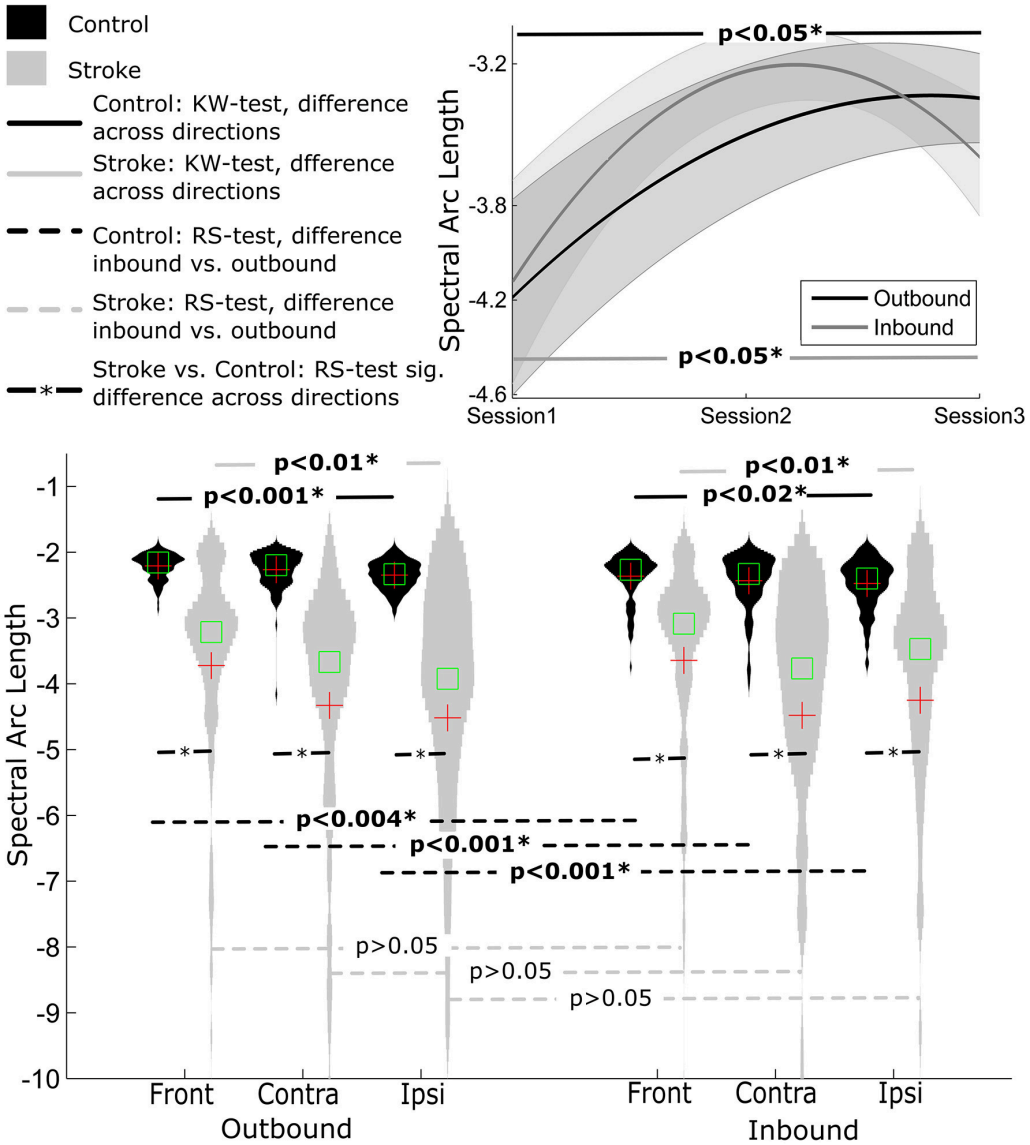
**Distribution of smoothness measure SAL for Control and Stroke participants in three directions for outbound and inbound movements. (Top-right)** Changes in smoothness across the three sessions (baseline, post-training and follow-up assessment).

Furthermore, time to peak velocity values were similar for each movement direction, regardless of movement segment, all *p*'s > 0.05 (see Figure 3).

#### Root mean square error

No significant difference was observed when comparing the outbound (1.52 ± 0.96) with the inbound (1.72 ± 1.32), all *p*'s > 0.1) movement segments. There was also a significant observable difference between movement directions for both inbound and outbound segments (both *p*'s < 0.001). *Post-hoc* Tukey's HSD test indicated that RMSE values were higher for outbound movements to the front direction (2.73 ± 1.64) than the contralateral and ipsilateral movement directions (0.96 ± 0.60 and 0.86 ± 0.63, respectively), *p* < 0.001. A similar pattern was revealed for inbound movements, with larger RMSE values for movements to the front direction (3.17 ± 2.23), compared to the contralateral and ipsilateral movement directions (1.04 ± 1.01 and 0.94 ± 0.74, respectively), *p* < 0.001.

### Differences between control and stroke participants

#### Spectral arc length

Overall, control participants made smoother movements than stroke patients, irrespective of movement direction and movement segment (all *p*'s < 0.001) (see Figure 3).

#### Normalized time to peak velocity

**Figure 4:**
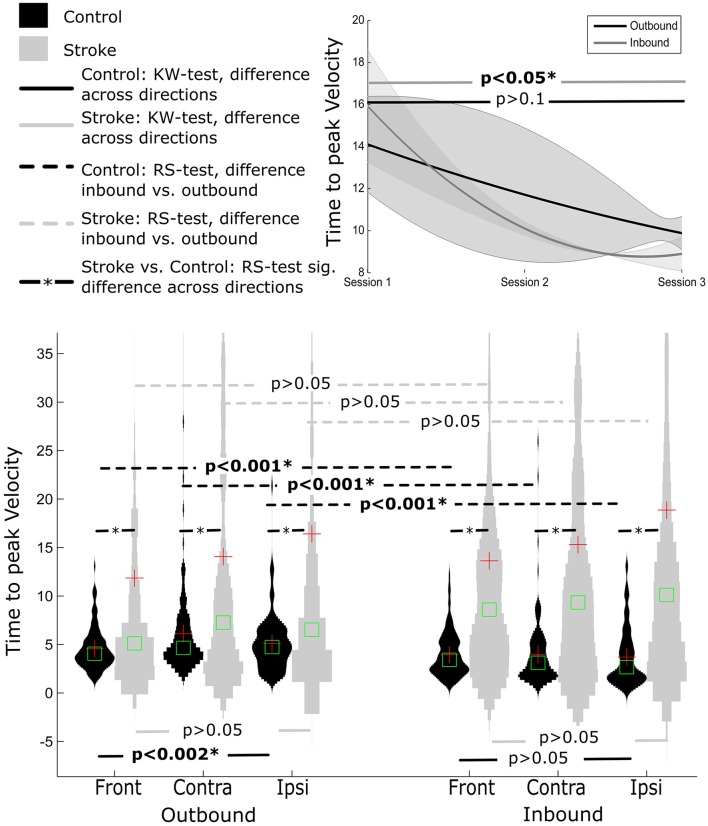
**Distribution of time to peak velocity measure distance normalized for Control and Stroke participants in three directions for outbound and inbound movements**. Changes in time to peak velocity across the three sessions (baseline, post-training and follow-up assessment).

Similarly, normalized time to peak velocity values were shorter for control participants compared to stroke participants, irrespective of movement direction and movement segment (all *p*'s < 0.001), Figure 4.

#### Root mean square error

**Figure 5:**
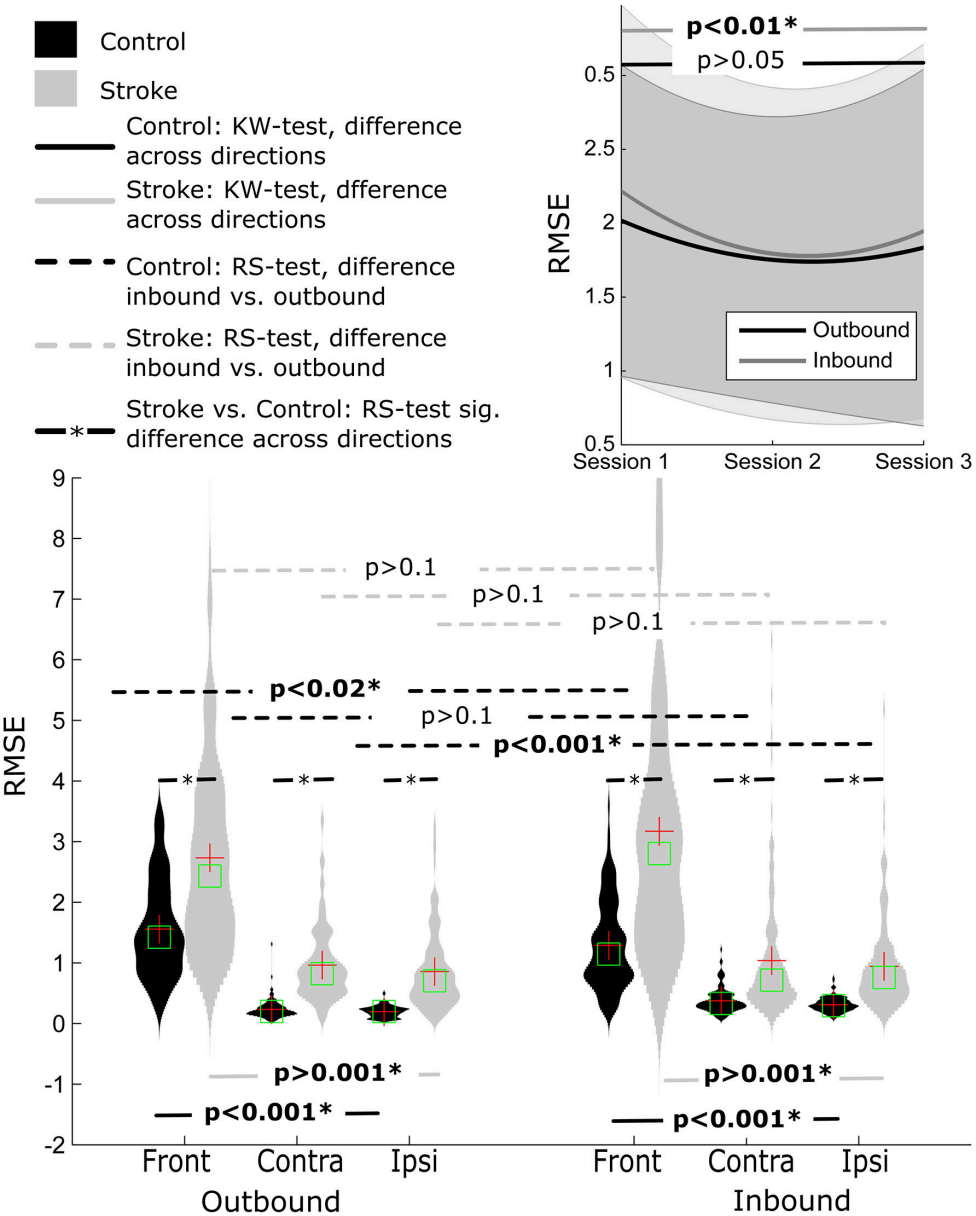
**Distribution of RMSE for Control and Stroke participants in three directions for outbound and inbound movements**. Changes in RMSE across the three sessions (baseline, post-training and follow-up assessment).

This was also true for RMSE values. The values were significantly lower for healthy control participants compared to stroke participants (all *p*'s < 0.001), Figure 5.

### Differences over sessions in stroke patients

#### Spectral arc length

There was a significant improvement in movement smoothness across sessions for inbound movements, *p* < 0.05. *Post-hoc* Tuckey's HSD test revealed a significant difference between pre-training (−4.13±2.32) compared to both post-training (−3.23±1.40) and follow-up (−3.59±2.16) assessment, *p* < 0.001. Movement smoothness also improved across time for outbound movements (pre-training = −4.19±2.26, post-training = −3.50±1.93, follow-up = 3.34 ± 1.47), but this improvement tended to diminish for the outbound segment in follow-up assessment (assessed using *Post-hoc* Tuckey test).

#### Normalized time to peak velocity

For inbound movements, there was a significant improvement in time to peak velocity across sessions (*p* < 0.05), with smaller time to peak velocity values at follow up (8.90 ± 9.21) compared to pre-training and post-training (15.98 ± 22.71 and 10.10 ± 14.50, respectively).

There was a trend toward smaller time to peak velocity values across time for outbound movements (pre-training: 14.15 ± 25.63, post-training: 11.77 ± 41.04, follow-up: 9.89 ± 15.46), however this effect failed to reach statistical significance (*p* > 0.6). Closer analysis of the data indicated that outbound segment time to peak velocity values were significantly different for 9 out of 12 participants, assessed using KW test.

#### Root mean square error

There was an improvement in RMSE across time for inbound movements (*p* < 0.01). *Post-hoc* analysis indicated that RMSE decreased between pre-training and post-training (1.68 ± 1.77 and 1.30 ± 1.37, respectively), but not between pre-training and follow-up (1.45 ± 1.45). No significant difference was observed for outbound movement although the pattern of improvement in RMSE showed a similar trend.

## Discussion

This preliminary study focused on investigating variations in kinematic task performance parameters (smoothness, temporal variability and task specific errors) for self-paced path reaching as a function of neurological status, direction, movement segment, and time using a portable, planar, robotic-manipulator (H-Man). Overall, movements performed by stroke participants were less smooth (and more variable), featured longer normalized time to peak velocities, and larger RMSE errors, compared to their age-matched neurologically healthy counterparts (Balasubramanian et al., 2012a). Furthermore, there were observable significant changes in performance over time for stroke patients using H-Man, compared to conventional scales which did not detect changes between initial and post-assessments.

### Direction affects performance for self-paced reaching-a potential indicator for direction specific training

Within the control group, the outbound reaching movement segments were generally smoother and featured shorter (with less variability) normalized time to peak velocity values when compared to inbound movements. In contrast, the assessed performance metrics for stroke participants were generally similar for outbound and inbound segments of movements. However, in both groups (control and stroke) significant directional differences (contralateral, front, and ipsilateral) were observed for SAL and Time to Peak Velocity, except deviation fit RMSE. In general, for both cases less variability and higher performance were observed for the front direction compared to ipsilateral and contralateral directions, implying ease in performing tasks in the front direction. These results are incongruent with those reported by Levin (1996) in which participants exhibited difficulties in performing reaching tasks in the front direction. However, in the Levin (1996) study the distance was fixed (point to point reach compared to self-paced maximum reach) and participants potentially had to cover a larger distance beyond the comfortable range which could induce this effect. Overall, the results are in accordance with previous studies which demonstrate task dependence weakness in the performance of stroke patients (Beer et al., 1999; Sukal et al., 2007).

This also gives an indication that performing the task in the ipsilateral and contralateral directions might be more useful compared to front direction (for self-paced tasks) when training due to the relative difficulty in performing smooth tasks in those directions. The recent study by Panarese et al. elucidating the automatized selection of motor task (direction) for the rehabilitative treatment is an example of a step in this direction (Panarese et al., 2012).

### Quantitative measures are sensitive to changes occurring during a session

While results of the clinical assessment indicated that there was no significant change for any of the measured variables, the kinematic parameters used in the current study were generally sensitive to changes in the performance of stroke participants across the three assessment time points. When reaching movements were quantified using kinematic parameters, it was observed that reaching movements were smoother and had lower task error values at post- compared to pre-training indicating a significant improvement in performance. These results are promising and lead to the hypothesis that these effects would be amplified if rehabilitation training continued. Despite improvements in kinematic performance immediately after training, these improvements were not maintained at 2-week follow-up. This is congruent with prior research (Brewer et al., 2007; Kwakkel et al., 2008; Norouzi-Gheidari et al., 2012) demonstrating that consistent and continued practice is required in order to offset decreases in motor function.

It is important to emphasize here that the main purpose of the study was not to investigate the efficacy of robotic rehabilitation over more conventional approaches, but to investigate feasibility of H-Man as a robotic assessment tool for tracking changes in performance. Nonetheless, the observed improvements in arm function are a positive step forward in the development of the H-Man robotic device, and future studies will continue to explore its efficacy for the rehabilitation of neurological disorders. First and foremost, data will be collected from a larger number of neurologically impaired and healthy individuals. As more data is collected, a database of normative motor function specific to the H-Man assessment can be formed, which would provide clinicians with the opportunity to compare a patient's performance against the reference values and make appropriate treatment decisions based on that data. Moreover, a delayed intervention randomized control trial (RCT) is currently underway that will reliably determine whether training with the H-Man device improves long-term post-stroke upper extremity function. Second, given the ample evidence demonstrating brain remodeling and plasticity as a consequence of learning, memory (Kolb and Gibb, 2014) and brain or peripheral injuries (Dimyan and Cohen, 2011; Nudo, 2013), future studies should employ neuroimaging and neurophysiological techniques in order to elucidate whether robotic training can result in neuroplasticity. Last, it would be most useful to examine how other aspects of voluntary goal-directed movement are affected by neurological insult, and how we might use robotic devices to assist in the recovery of these functions. For example, it would certainly be worthwhile examining the ability to cancel pending actions (Logan et al., 1984; Logan, 1994). To date, research into the volitional inhibitory control of manual motor actions and the ability to choose between alternative actions to achieve an identified goal have been mostly neglected by scientists working either on rehabilitation and on BCI (Mirabella, 2012). This is unfortunate given that volitional inhibition is a fundamental function of behavioral flexibility that allows us to quickly adapt behavior to unattended changes either in our thoughts or in the external environment (Mirabella, 2014), and involved both the pre-motor and the primary motor cortices (Mirabella et al., 2011; Mattia et al., 2012, 2013).

## Author contributions

The conception and the development of the study protocol were carried out by: AH, AB, CH, YL, CK, LX, KC, EB, and DC. The hardware and software setup was designed by: AH, AB, and WD. Clinical study and data from healthy and stroke patients were collected by: CK, KC, CH, AB, DV, LY, and WD. Data analysis was done by: AH, AB, KC, and LY. The manuscript was written by: AH, AB, and CH. All the authors read and approved the final manuscript.

### Conflict of interest statement

The authors declare that the research was conducted in the absence of any commercial or financial relationships that could be construed as a potential conflict of interest.
